# The influence of integrated geriatric outpatient clinics on the health care utilization of older people

**DOI:** 10.1186/s12877-020-01782-7

**Published:** 2020-10-02

**Authors:** Yu-Ju Wei, Cheng-Fang Hsieh, Yu-Ting Huang, Ming-Shyan Huang, Tzu-Jung Fang

**Affiliations:** 1 Division of Geriatrics and Gerontology, Department of Internal Medicine, Kaohsiung Medical University Hospital, Kaohsiung Medical University, Kaohsiung, Taiwan; 2 Division of Medical Statistics and Bioinformatics, Department of Medical Research, Kaohsiung Medical University Hospital, Kaohsiung Medical University, Kaohsiung, Taiwan; 3 E-Da Cancer Hospital, Kaohsiung, Taiwan; 4 School of Medicine, I-Shou University, Kaohsiung, Taiwan; 5 Graduate Institute of Clinical Medicine, College of Medicine, Kaohsiung Medical University, Kaohsiung, Taiwan

**Keywords:** Geriatric integrated outpatient clinic, Older people, Health care utilization, Comprehensive geriatric assessment

## Abstract

**Background:**

The number of people aged greater than 65 years is growing in many countries. Taiwan will be a superaged society in 2026, and health care utilization will increase considerably. Our study aimed to evaluate the efficacy of the geriatric integrated outpatient clinic model for reducing health care utilization by older people.

**Methods:**

This was a retrospective case-control study. Patients aged greater than 65 years seen at the geriatric outpatient clinic (Geri-OPD) and non-geriatric outpatient clinic (non-Geri-OPD) at a single medical centre were age and sex matched. Data on the number of outpatient clinic visits, emergency department visits, hospitalizations and medical expenditures were collected during the first and second years. A subgroup analysis by Charlson comorbidity index (CCI) and older age (age≧80 years) was performed, and the results were compared between the Geri-OPD and non-Geri-OPD groups.

**Results:**

A total of 6723 patients were included (3796 women and 2927 men). The mean age was 80.42 ± 6.39 years. There were 1291 (19.2%) patients in the Geri-OPD group and 5432 (80.8%) patients in the non-Geri-OPD group. After one year of regular follow-up, the Geri-OPD patients showed a significant reduction in the types of drugs included in each prescription (5.62 ± 10.85) and the number of clinic visits per year (18.18 ± 48.85) (*P* < 0.01). After a two-year follow-up, the number of clinic visits, emergency department visits, and hospitalizations and the annual medical costs were still decreased in the Geri-OPD patients. The Geri-OPD patients had more comorbidities and a higher rate of health care utilization than the non-Geri-OPD patients. In the subgroup analysis, patients with more comorbidities (CCI≧2) and an older age (≧80 years) in the Geri-OPD group showed a significant reduction in health care utilization. The Geri-OPD patients also showed a significant decrease in medical utilization in the second year compared with the non-Geri-POD patients.

**Conclusion:**

The Geri-OPD reduced medical costs, the number of drugs prescribed, and the frequency of outpatient clinic visits, emergency department visits and hospitalizations in older patients with complicated conditions. The effect was even better in the second year.

## Background

People aged 65 years or older are the world’s fastest-growing group, according to the United Nations’ report in 2019 [1]. Sixty-six percent of the increase in the older population between 2015 and 2050 will occur in Asia [2]. The ageing process leads not only to physical impairment but also to mental and social problems. It results in increased dependency and greater spending on health care [3]. Age-associated chronic disorders, such as dementia and cardiovascular diseases, account for most of the disease burden, leading to enormous costs in higher income countries [4, 5]. Polypharmacy resulting from multiple comorbidities is also an important issue in older people. It is not only an economic burden but also a risk factor for mortality and morbidity [6]. The definition of polypharmacy is variable. In general, polypharmacy refers to excessive unnecessary drug consumption or the use of high numbers of drugs and is a convenient evaluation method to use in practice [7, 8]. Older adults using more than 8 drugs have increased risks of rehospitalization [9]. Higher comorbidities are associated with increased risks of adverse drug events, which results in a vicious cycle [10].

Frail older people with multi-morbidity have a greater chance of receiving fragmented medical care, which is associated with unnecessary medical utilization and increased medical costs [11–13]. In recent years, some programmes, such as the Program of Research to Integrate Services for the Maintenance of Autonomy (PRISMA) in Canada and the Health and Welfare Information Portal (ZWIP) study in the Netherlands, have aimed to integrate health care and social services for frail older people [13, 14]. A decrease in functional decline and fewer emergency room visits were obtained after 3 years in the PRISMA study, for which the coverage rate reached 70% [14]. Barriers to integrated care, such as funding, leadership, time constraints, care system complexity and shared values, could affect the outcomes [14].

Taiwan is aging at a very high speed. Taiwan became an aging society in 1993; it became an aged society in 2018 and will be a superaged society in 2026 [3]. More than two-thirds of older people have one chronic disease. Approximately 5% of men and 18% of women in Taiwan have 3 or more chronic diseases [15]. The medical fees for people aged older than 65 years increased by 7.5% every year from 1998 to 2006 in Taiwan [15]. In 2007, older adults in Taiwan comprised 10.1% of the population but accounted for 36.2%, approximately 512 billion New Taiwan dollars (NTD), of yearly medical costs [15]. Polypharmacy is frequent in the Taiwanese aged population; 81.1% had received more than 5 prescriptions, and 38.1% had major polypharmacy (i.e., more than 10 medications) [16]. Taiwan’s 2016–2060 population projections disclosed that the birth rate decreased, and the country will have negative population growth in 2021 but an increased proportion of aged groups. The medical burden has grown rapidly with the aging speed in Taiwan. In addition, the dependency ratio will rise from 36 out of every 100 persons of working age in 2016 to 93 out of 100 by 2060. The trend of an increased proportion of older people and decreased population growth in Taiwan is similar to that described in the 2015 United Nations’ report. The report showed that the population aged greater than 60 years was the fastest growing group but that the global population will reach a negative growth rate [2, 3]. Taiwan is located in East Asia, and its traditional concepts include respecting and caring for parents and grandparents. Therefore, the caregiver burden will increase along with the elevated dependency rate. In Taiwan’s health insurance system, patients are not limited to seeking medical services from specialists in the hospital and do not need a referral from primary care physicians. However, demanding medical resources and fragmented medical services have resulted in rapidly growing burdens in terms of outpatient department (OPD) visits and medical costs. Therefore, integrated medical care is important to reduce unnecessary medical utilization and rising medical costs in a rapidly aging society, and we have established geriatric integrated outpatient clinics for older patients who are frequent users of outpatient services. The establishment of an integrated clinic in the hospital was supported by a programme of the National Health Insurance Administration.

**Geriatric integrated outpatient clinic at KMUH (**Kaohsiung Medical University Hospital): The geriatricians at the clinic provided patients with individualized care plans after performing a comprehensive geriatric assessment. The comprehensive geriatric assessment (CGA) is defined as “a multidimensional and multidisciplinary process that identifies medical, social and functional needs and develops an integrated multidisciplinary care plan to meet the need” [17]. Psychiatrists, doctors of rehabilitation and physical medicine and neurologists can be consulted at the same time. The geriatrician integrates the medicine and the treatment plans. The pharmacist, the dietitian and the social worker provide recommendations when they are consulted in the clinic. The health educator provides health education and connects the patients to social resources and long-term care services as needed. The patients are transferred from other doctors because of their polypharmacy or high utilization of medical care, such as consulting 3 or more physicians for their care or experiencing frequent emergency room visits or repeated hospitalizations. Other patients seek out the clinic on their own because they want to receive integrated care for similar problems. The medical fee is paid by the National Health Insurance. The geriatric integrated clinic is promoted in the hospital to doctors and patients via posts and an electronic system.

Hospital inpatients who are older than 55 years are the major beneficiaries of the CGA in terms of mortality, daily living activities and independence [17]. However, the effect of the CGA in the clinic on health care utilization is less often reported. In this study, we evaluated the effect of geriatric integrated outpatient clinics on reducing health care utilization in older people.

## Methods

We enrolled patients from the geriatric integrated outpatient clinic at Kaohsiung Medical University Hospital (KMUH), a tertiary medical centre, from January 1, 2013, to July 31, 2016, as the target subjects **(the Geri-OPD group)**. All patients were aged older than 65 years. These patients received regular outpatient clinic follow-up. “Regular follow-up” was defined as at least four service claims annually for ambulatory or outpatient clinics because the longest possible prescription in our insurance system was 3 months. The matched control group **(the non-Geri-OPD group)** for this study was also extracted from the KMUH Research Database (KMUHRD). Patients in this control group were also older than 65 years and had at least four service claims in the Department of Internal Medicine or the Division of Family Medicine or Neurology for similar medical conditions. We randomly selected 5432 control subjects (4 for every target patient) who were matched with the study group in terms of age and sex by the electronic system. Chart numbers were used to avoid duplication in both groups. In our study, polypharmacy was defined as more than 8 kinds of long-term drugs used for chronic disease control. The primary outcomes were the number of outpatient clinic visits, the number of medication prescriptions and cost. Secondary outcomes were the number of hospitalizations and emergency room visits. Patients who received renal replacement therapy before visiting the geriatric integrated outpatient clinic and patients with malignancy were not included because these diseases require high medical resource utilization that is difficult to reduce with a geriatric integrated OPD.

The KMUHRD includes data for approximately 800,000 patients who attended KMUH from 2009 to 2016. The KMUHRD offers a comprehensive database with coverage of ambulatory care, hospital admissions, dental services, drug dispensation records, and biochemical data. All diagnoses are coded according to the International Classification of Diseases, 9th Revision, Clinical Modification (ICD-9-CM). The database is managed by the Division of Medical Statistics and Bioinformatics of KMUH. To protect the confidentiality of the study participants and to comply with the Personal Information Protection Act, all personal identifiers have been removed, and only authorized researchers were permitted to perform data linkage, processing and statistical analyses with specified computers in a separate room with 24-h monitoring using encrypted identifiers. All data analysts were required to sign a confidentiality agreement. Tables and figures from the statistical analysis were permitted to be released only after inspection by managing personnel. This study was performed after obtaining the approval of the institutional review board of KMUH.

Comparisons were performed between the target study group and the matched control group. The baseline demographics of these two groups were described in terms of sex, age, comorbidities, Charlson comorbidity index (CCI), number of hospitalizations, number of outpatient clinic visits, number of emergency department visits, and medical cost in the year before the index date. We performed 6-month, one-year and 2-year analyses for these 2 groups for number of hospitalizations, outpatient clinic visits, emergency department visits, and medical expenditures. A subgroup analysis with the CCI classification was performed to evaluate the efficacy of integrated outpatient clinics for the oldest population and for high comorbidity patients (CCI ≥ 2). A CCI score greater than 2 or 3 was defined as a high comorbidity score and is related to high mortality [18–22].

The chi-square test was used to compare the distribution of sociodemographic characteristics and comorbidity between the two groups. Data are expressed as percentages or the mean ± standard deviation for patient medical utilization estimates, and the independent t-test was used for continuous variables. Medical utilization was calculated 1 year before and 6 months, 12 months and 24 months after the index visit for analysis. Medical costs in NTD were expressed after logarithmic transformation of the original values. All data processing and statistical analysis were performed with SAS 9.4 software (Cary, NC, USA).

## Results

A total of 6723 patients were enrolled; 1291 (19.2%) patients were from the Geriatric Integrated Outpatient Clinic (the Geri-OPD group), and 5432 (80.8%) patients were not from the clinic (the non-Geri-OPD group). The mean age was 80.42 ± 6.39 years, and 56.46% were women. In the population older than 80 years, the mean age was 85.67 ± 4.17 years. In the CCI ≥ 2 population, the mean age was 81.61 ± 6.28 years.

At baseline, the Geri-OPD group had significantly more comorbidities, including congestive heart failure (6.6%), peripheral vascular disease (1.7%), pulmonary disease (10.8%), peptic ulcers (11.7%), diabetes mellitus (19.2%), diabetes mellitus complications (10.5%), and depression (4.5%). In the subgroup analysis of the patients aged greater than 80 years, the Geri-OPD group had a higher percentage of congestive heart failure, pulmonary disease, diabetes mellitus and depression than the non-Geri-OPD group. Among the high comorbidity patients (CCI ≥ 2) in the Geri-OPD group, cerebrovascular accidents, diabetes mellitus and renal disease accounted for higher percentages of the comorbidities. The Geri-OPD patients had significantly higher medical utilization than the non-Geri-OPD patients, a finding that was also the case for the age≧80 and CCI≧2 subgroups. For instance, Geri-OPD patients had 66.40 ± 51.45 OPD visits every year and non-Geri-OPD patients had 35.86 ± 48.53 outpatient visits per year (*P* < 0.0001). The median interquartile range (IQR) of OPD visits among every Geri-OPD patient every year was 67, and in non-Geri-OPD patients, it was 56 (*P* < 0.0001). The Geri-OPD patients with a CCI ≥ 2 took 24.05 ± 4.86 kinds of drugs, and non-Geri-OPD patients took 21.69 ± 10.67 kinds of drugs on average (*P* < 0.0001), indicating severe polypharmacy. The annual outpatient care cost of the Geri-OPD group and the non-Geri-OPD group was 507.7 ± 408.0 (log NTD) and 269.1 ± 403.2 (log NTD), respectively (*P* < 0.0001). The median IQRs of annual outpatient care costs were 522.55 (log NTD) and 412.06 (log NTD) for the Geri-OPD and non-Geri-OPD groups, respectively (*P* < 0.0001) (Table 1, Additional file Table S1 and Table S2).

**Table 1:** Basic patient demographics

	Total	Geri-OPD	Non-Geri-OPD	*P*
Number, n (%)	6723 (100)	1291 (19.2)	5432 (80.8)	
Sex				
Female, n (%)	3796 (56.46)	737 (57.1)	3059 (56.3)	0.61
Male, n (%)	2927 (43.53)	554 (42.9)	2373 (43.7)	
Age, mean ± standard deviation (SD)	80.42 ± 6.39	80.90 ± 6.42	80.71 ± 6.38	0.33
65–80 years, n (%)	3095 (46.04)	579 (44.8)	2516 (46.3)	0.34
≥ 80 years, n (%)	3628 (53.96)	712 (55.2)	2916 (53.7)	
Charlson comorbidity index (CCI)				< 0.01
CCI = 0, n (%)	2993 (44.5)	462 (35.8)	2531 (46.6)	
CCI = 1, n (%)	1404 (20.9)	300 (23.2)	1104 (20.3)	
CCI ≥ 2, n (%)	2326 (34.6)	529 (41)	1797 (33.1)	
Medical problems, n (%)				
Acute myocardial infarction	228 (3.4)	42 (3.3)	186 (3.4)	0.76
Congestive heart failure	442 (6.6)	111 (8.6)	331 (6.1)	< 0.01
Peripheral vascular disease	111 (1.7)	31 (2.4)	80 (1.5)	0.02
Cerebral vascular accident	1353 (20.1)	261 (20.2)	1092 (20.1)	0.93
Dementia	747 (11.1)	154 (11.9)	593 (10.9)	0.30
Pulmonary disease	725 (10.8)	264 (20.4)	461 (8.5)	< 0.01
Connective tissue disorder	45 (0.7)	12 (0.9)	33 (0.6)	0.20
Peptic ulcer	784 (11.7)	191 (14.8)	593 (10.9)	0.00
Liver disease	175 (2.6)	40 (3.1)	135 (2.5)	0.21
Diabetes mellitus	1288 (19.2)	318 (24.6)	970 (17.9)	< 0.01
Diabetes mellitus complications	595 (8.9)	135 (10.5)	460 (8.5)	0.02
Renal disease	741 (11.0)	157 (12.2)	584 (10.8)	0.15
Depression	304 (4.5)	77 (6)	227 (4.2)	0.01
Annual outpatient department visits (mean ± SD)		66.40 ± 51.45	35.86 ± 48.53	< 0.01
Annual emergency room visits (mean ± SD)		2.36 ± 6.86	0.968 ± 3.27	< 0.01
Number of drugs (mean ± SD)		23.22 ± 5.6	16.34 ± 12.43	< 0.01
Annual hospitalizations (mean ± SD)		0.57 ± 1.05	0.23 ± 0.69	< 0.01
Length of hospital stay (days/year)		6.94 ± 16.88	2.48 ± 9.19	< 0.01
Cost of each clinic visit (log, mean ± SD, NTD)		7.41 ± 1.07	5.05 ± 3.44	< 0.01
Cost of each hospitalization (log, mean ± SD, NTD)		3.58 ± 5.11	1.66 ± 3.92	< 0.01
Cost of annual outpatient care (log, mean ± SD, NTD)		507.7 ± 408.0	269.1 ± 403.2	< 0.01
Cost of annual hospitalizations (log, mean ± SD, NTD)		6.15 ± 11.39	2.52 ± 7.46	< 0.01

Abbreviation: *NTD* New Taiwan dollars

**Fig. 1:**
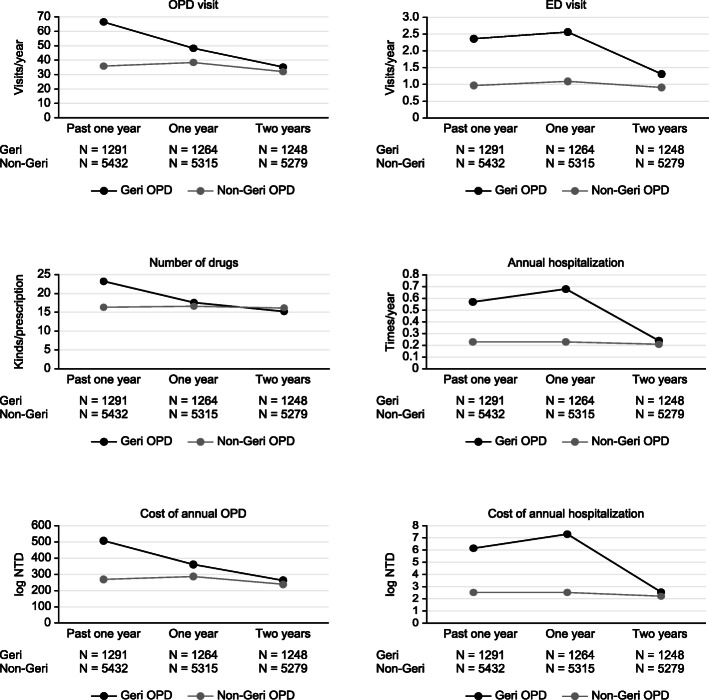
High health care utilization decreased significantly in Geri-OPD patients

**Fig. 2:**
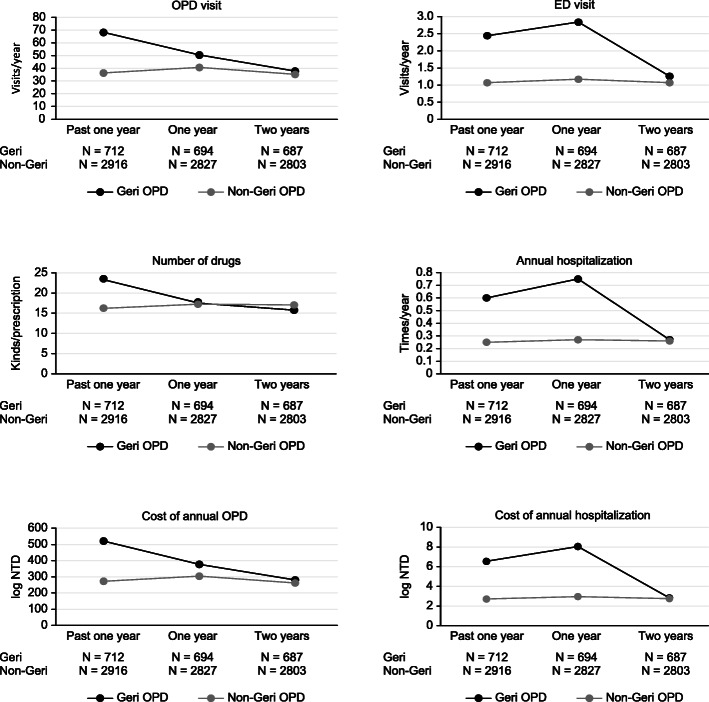
Health care utilization also decreased significantly in Geri-OPD patients aged ≥80 years

**Fig. 3:**
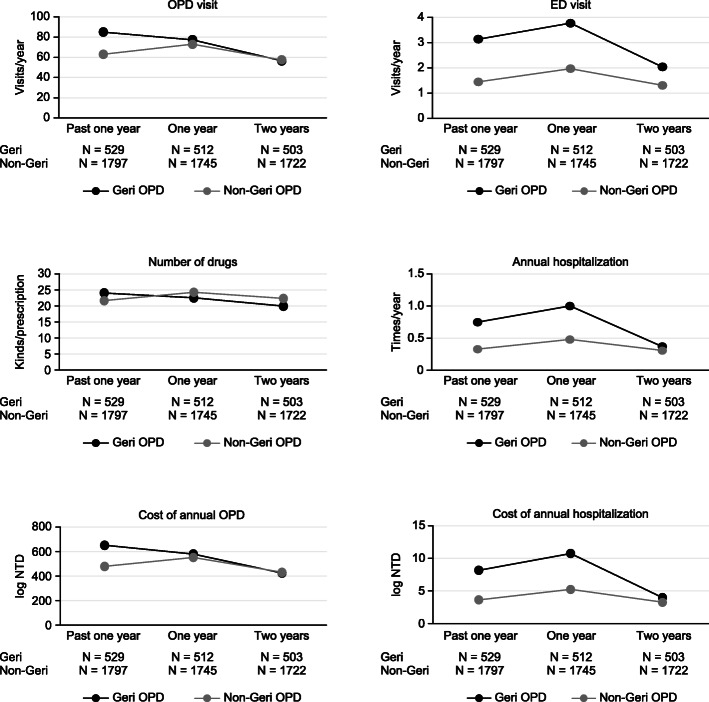
The reduction in health care utilization was obvious in the Geri-OPD patients with a CCI ≥ 2

At the one-year follow-up, the Geri-OPD patients had a reduction of 5.62 ± 10.85 drugs among the kinds of drugs used; in comparison, the non-Geri-OPD patients had 0.30 ± 10.51 more drugs used in every prescription (*P* < 0.0001). The number of outpatient clinic visits was reduced by 18.18 ± 48.85 visits per year (*P* < 0.0001), and the annual cost of outpatient visits also decreased for the Geri-OPD patients. However, the number of annual hospitalizations (times/year), the annual length of hospital stays (days/year) and the cost of each hospitalization (log NTD) increased significantly among the Geri-OPD patients (0.11 ± 1.24, 1.60 ± 19.64, and 1.13 ± 6.54, respectively) (Fig. 1, Table 2 and Table 3). In the subgroup analysis of the patients aged older than 80 years, patients treated at geriatric clinics had a reduction of 17.75 ± 51.94 visits among annual outpatient visits, whereas the patients treated at non-geriatric clinics had 4.33 ± 30.66 more annual outpatient visits (*P* < 0.0001). The Geri-OPD patients were prescribed 5.79 ± 10.87 fewer drugs, but the non-Geri-OPD patients were prescribed 1.02 ± 10.44 more drugs (*P* < 0.0001). The Geri-OPD patients had decreased medical costs at each ambulatory care visit but higher medical expenditures for hospitalization (Fig. 2, Additional file Table S3 and Table S5). Among the CCI ≥ 2 patients, the Geri-OPD patients had a decrease in the number of annual outpatient visits by 7.56 ± 55.95 visits, while the non-Geri-OPD patients had an annual increase in outpatient visits of 10.04 ± 39.85 visits (*P* < 0.0001). The Geri-OPD patients were prescribed 1.49 ± 7.34 fewer medications, while the non-Geri-OPD patients an increase of 2.58 ± 8.96 drugs among the number of drugs prescribed was noted (*P* < 0.0001). The Geri-OPD patients had decreased medical costs at each outpatient visit, while the non-Geri-OPD patients had increased medical expenditures (*P* = 0.0005). Both the Geri-OPD and non-Geri-OPD patients had increased hospitalization expenditures and lengths of hospital stays (Fig. 3, Additional file Table S4 and Table S6).

**Table 2:** Health care utilization of all patients

	First year			Second year		
	Geri-OPD (*N* = 1291)	Non-Geri-OPD (*N* = 5432)	*P*	Geri-OPD (*N* = 1264)	Non-Geri-OPD (*N* = 5315)	*P*
Annual outpatient department visits (mean ± SD)	48.22 ± 54.95	38.36 ± 49.43	< 0.01	35.17 ± 50.02	32.05 ± 44.80	0.04
Annual emergency room visits (mean ± SD)	2.56 ± 6.29	1.09 ± 3.84	< 0.01	1.31 ± 3.76	0.91 ± 3.82	0.01
Number of drugs (mean ± SD)	17.59 ± 10.96	16.63 ± 12.37	0.01	15.23 ± 12.43	16.14 ± 12.54	0.02
Annual hospitalizations (mean ± SD)	0.68 ± 1.01	0.23 ± 0.68	< 0.01	0.24 ± 0.60	0.21 ± 0.63	0.10
Length of hospital stay (days/year)	8.54 ± 16.46	2.63 ± 10.27	< 0.01	2.45 ± 8.30	2.16 ± 8.67	0.25
Cost of each clinic visit (log NTD, mean ± SD)	6.10 ± 2.88	5.13 ± 3.39	< 0.01	4.80 ± .351	4.91 ± 3.47	0.32
Cost of each hospitalization (log NTD, mean ± SD)	4.72 ± 5.36	1.63 ± 3.87	< 0.01	1.86 ± 4.06	1.48 ± 3.70	0.01
Cost of annual outpatient care (log NTD, mean ± SD)	360.9 ± 435.6	286.8 ± 402.4	< 0.01	262.8 ± 401.2	239.0 ± 361.1	0.05
Cost of annual hospitalizations (log NTD, mean ± SD)	7.31 ± 10.85	2.52 ± 7.43	< 0.01	2.55 ± 6.46	2.22 ± 6.80	0.10

**Abbreviation:** *NTD* New Taiwan dollars

**Table 3:** Change in the health care utilization of all patients

	First year			Second year		
	Geri-OPD (*N* = 1291)	Non-Geri-OPD (*N* = 5432)	*P*	Geri-OPD (*N* = 1264)	Non-Geri-OPD (*N* = 5315)	*P*
Annual outpatient department visits (mean ± SD)	−18.18 ± 48.85	2.50 ± 28.72	< 0.01	−13.05 ± 33.68	−6.31 ± 34.74	< 0.01
Annual emergency room visits (mean ± SD)	0.20 ± 6.49	0.11 ± 3.96	0.64	−1.25 ± 6.26	− 0.18 ± 4.80	< 0.01
Number of drugs (mean ± SD)	−5.62 ± 10.85	0.30 ± 10.51	< 0.01	− 2.37 ± 9.22	−0.49 ± 10.05	< 0.01
Annual hospitalizations (mean ± SD)	0.11 ± 1.24	0.00 ± 0.88	0.01	−0.44 ± 1.04	−0.03 ± 0.84	< 0.01
Length of hospital stay (days/year)	1.60 ± 19.64	0.16 ± 12.69	0.01	− 6.09 ± 17.22	−0.48 ± 12.15	< 0.01
Cost of each clinic visit (log NTD, mean ± SD)	−1.31 ± 2.93	0.08 ± 3.38	< 0.01	−1.30 ± 3.40	− 0.22 ± 3.38	< 0.01
Cost of each hospitalization (log NTD, mean ± SD)	1.13 ± 6.54	−0.03 ± 5.05	< 0.01	− 2.86 ± 6.11	−0.15 ± 4.92	< 0.01
Cost of annual outpatient care (log NTD, mean ± SD)	− 146.8 ± 393.4	17.63 ± 236.4	< 0.01	−98.08 ± 264.1	− 47.80 ± 279.3	< 0.01
Cost of annual hospitalizations (log NTD, mean ± SD)	1.16 ± 13.36	− 0.01 ± 9.59	0.01	−4.76 ± 11.17	−0.30 ± 9.12	< 0.01

**Abbreviation:** *NTD* New Taiwan dollars

**Fig. 4:**
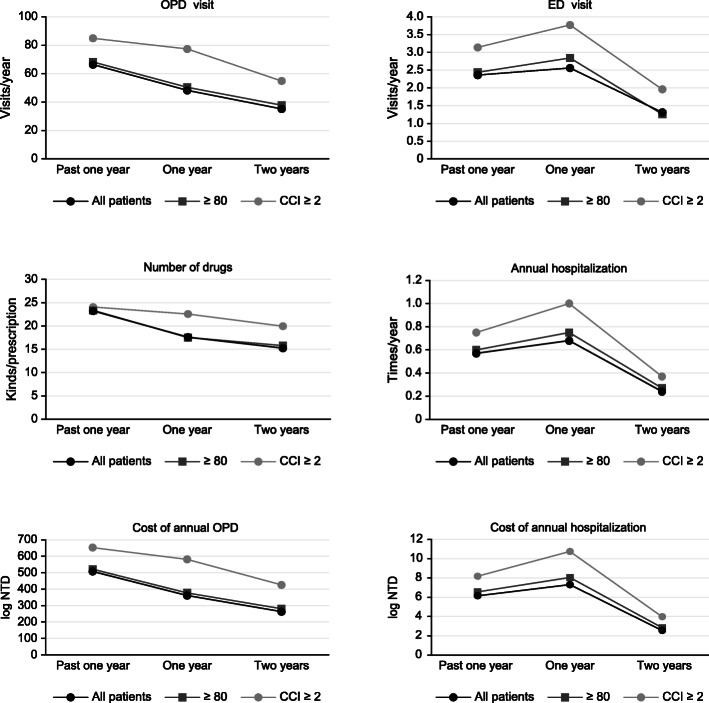
Health care utilization decreased gradually over 2 years of Geri-OPD follow-up

At the two-year follow-up, in the Geri-OPD group, the number of outpatient visits, drugs prescribed, emergency department visits, and hospitalizations and the costs were significantly reduced compared to those in the non-Geri-OPD group. The effect was the same for the subgroups of patients older than 80 years and those with a CCI ≥ 2. The patients with higher comorbidities (CCI≧2) had much higher medical expenditures; however, the older population (age≧80 years) had medical utilization similar to that of the whole group of patients (Fig. 4).

In summary, this retrospective study revealed that the geriatric integrated outpatient clinic reduced the annual medical costs of outpatient care because it was associated with a decrease of 18 outpatient visits within 1 year and an average decrease of 5 kinds of medications for every prescription.

## Discussion

The integrated care model of our geriatric ambulatory clinic did reduce the frequency of outpatient visits and the number of drugs prescribed and thus the total annual cost of outpatient care. However, medical expenditures associated with hospitalization increased in the first year for the Geri-OPD patients compared with the non-Geri-OPD patients. In the second year, ambulatory clinic visits, emergency department visits, the frequency of hospitalizations and cost decreased significantly compared with the first year of geriatric integrated outpatient clinic care. The Geri-OPD patients still had more outpatient and emergency visits and higher medical expenditures for every hospitalization than the non-Geri-OPD patients; however, the annual medical costs for outpatient care and hospitalization and the length of hospitalization did not significantly differ between these two populations after 2 years.

In this study, the dramatic decrease in the number of medical services (outpatient visits) and drugs prescribed reduced medical expenditures in the geriatric integrated outpatient clinic. The older patients who received geriatric integrated outpatient clinic care in our study had reduced health care utilization in the first year (Fig. 1), and this was reduced even further in the second year, which was better than the results in other reports in recent years [13, 14]. The overall proportion of patients with multiple doctor visits was as high as 39.4%, according to the National Health Insurance Research Database in Taiwan [23]. The top five reported diagnoses of older people treated at ambulatory care visits were diseases involving the circulatory, respiratory, musculoskeletal, and nervous systems and endocrine disorders [24]. Our geriatric integrated care model involved geriatricians, neurologists, psychiatrists and doctors in physical medicine and rehabilitation, social workers, health educators, dietitians and pharmacists. Thus, we could handle the most common problems of older adults who were frequent users of insurance. The CGA is an important part of our integrated care system at the outpatient clinic, especially in the evaluation of geriatric syndrome and patient-centred care. There is currently no standardized algorithm for acute and chronic care for older people [25].

The average number of outpatient clinic visits of older adults (older than 65 years) in Taiwan was reported to be 26.8 ± 22.7 (mean ± standard deviation) visits per year in 2004 [24]; the number in our study was 66.40 ± 51.45 for the Geri-OPD patients and 35.86 ± 48.53 in the control group. The number of outpatient clinic visits in our study was much higher than that in other reports [26–28]. The high health care utilization was related not only to how complicated the patients’ conditions were but also to the near-total coverage of medical fees by the national insurance system. The latter factor affected the patients’ health care-seeking behaviours. The most common comorbidities in patients with multiple physician visits in Taiwan are type 2 diabetes mellitus (3.68%) and hypertension (3.79%), according to the National Health Insurance Database [23]. In our study, the most common comorbidities were diabetes mellitus (19.2%) and cerebrovascular accidents (20.1%). Our patients had more complicated conditions than the average patient in Taiwan. The reduction in health care utilization was even more obvious in the second year of our study, which indicated that the treatment plans required time to take effect for older patients with complicated conditions. The trend was most obvious in the oldest patients in the population. The number of prescriptions actually increased in the first year, which may have been secondary to newly diagnosed problems, such as geriatric syndrome. Geriatric syndrome was frequently missed and was considered normal ageing before the implementation of the CGA. Therefore, increased health care utilization was needed in the first year to treat newly diagnosed medical problems and modify drug use. The use of the CGA in outpatient services has been less studied. One possible reason is that the CGA and subsequent formation of an individualized plan are time consuming. We tried to separate the CGA and the plan formulation process into the first 2 or 3 visits in our clinical practice, and this helped us to identify major and potential problems in an efficient way. According to a previous review article, the performance of the CGA in the clinic had no statistical effect on survival, but one recent randomized controlled trial showed a beneficial effect on frailty after 2 years in patients with very complicated conditions (age ≥ 75 years, ≥ 3 current diagnoses, and ≥ 3 hospitalizations during the 1 year prior to study inclusion) [29, 30]. Our study provides evidence that performing a CGA in the outpatient clinic for patients with complicated conditions can reduce their high health utilization for 2 years.

Health care utilization was reduced in the subgroup of Geri-OPD patients with a high number of comorbidities in our study (Fig. 3). Approximately 34% of the patients in our study had a high number of comorbidities (CCI ≥ 2). Higher numbers of comorbidities are correlated with an increased cost of hospitalizations and high economic burden, which was also found in our study. Librero reported that patients with more comorbidities have a longer length of hospital stay, higher mortality, and higher readmission rates [31]. A higher number of comorbidities is likely to lead to more complications during admission, which results in higher medical costs for each hospital stay. Multidisciplinary interventions can reduce hospital admissions and falls in older adults and increase patient satisfaction with health care services, but institutionalization and mortality rates might not decrease [32–34]. In our study, the subgroup of patients with more comorbidities had higher health care utilization than the subgroup of patients older than 80 years (Fig. 4). The high number of comorbidities was associated with high health care utilization in our study. However, the average age of all patients and of the two subgroups was older than 80 years, which may underestimate the effect of ageing on health care utilization.

Initially, the average number of medications used by our patients was more than 20. The number of drugs used by Geri-OPD patients after geriatric integrated outpatient clinic care was significantly less than that of non-Geri-OPD patients. Nevertheless, all of the patients in this study still took more than 15 kinds of medications, even after the second year. It was difficult to reduce the number of drugs used in patients with multiple comorbidities. Thus, we focused on the prevention of potentially inappropriate prescriptions. In several studies and reviews, polypharmacy and inappropriate prescribing had an adverse effect on older people due to the higher risk of falls and drug-related harm [35–37]. From 2001 to 2004, 19.1% of patients older than 65 years who were covered by Taiwanese National Health Insurance had an inappropriate medication prescription according to the Beers criteria [38]. Older people taking inappropriate medications have significantly more ambulatory care visits, emergency department visits and hospital admissions [38]. A prospective study including 6666 adults aged older than 50 years in Ireland revealed that polypharmacy (> 4 medications) was associated with the number of falls in older adults if antidepressants or benzodiazepines were included [39]. A previous study revealed that 50% of older adults take one or more medications that are unnecessary and that having a clinical pharmacist on the multidisciplinary team could help reduce drug numbers [40]. Polypharmacy could be attributable to the presence of multiple chronic diseases and to patients visiting multiple ambulatory clinics because Taiwan’s health insurance does not restrict health care utilization by any individual. In our geriatric outpatient integrated care system, unnecessary medications were discontinued after the treatment goal was set, and the patient’s functional status was considered. This intervention could slow down the vicious cycle of comorbidities, multiple ambulatory clinic visits, polypharmacy, emergency department visits, and hospitalizations.

An integrated care model for older people was discussed in 1983 by Albert and included acute care units, rehabilitation day hospitals, nursing homes, outpatient clinics, and home care [41]. Compared to traditional outpatient clinics, integrated outpatient clinics can decrease acute care utilization, reduce medical costs and decrease subspecialty clinic use, as was the case for the Collaborative Assessment and Rehabilitation for Elders (CARE) Program in the United States, which was designed for chronically ill older adults who did not meet the indications for inpatient rehabilitation [26–28, 42]. Geriatric evaluation can reduce functional decline without increasing medical cost [42–45]. It is difficult to efficiently develop appropriate guidelines for caring for older people with several comorbidities [46, 47]. In the preliminary data of one study that embedded geriatric service into primary care by providing on-site consultations with a geriatrician and geriatric nurse case manager, the mean number of subspecialty clinic visits (7.4 ± 9.8) declined significantly during the first year after enrolment and after the second year [26]. Fragmented care was evident in that study. In our efficient geriatric integrated care model, which was provided in the outpatient department of the hospital, we reduced the health care utilization of the oldest group of patients, who had complicated conditions and needed a very high number of clinic visits per year.

Integrated health care for older adults is currently an important issue and will continue to be important in the future. Families and well-trained caregivers are an important part of a comprehensive integrated care system. Geriatric clinics that include a range of health services can provide older patients with more convenient medical services [48]. The health care expenditures of healthier older people are similar to those of less healthy people despite their longer life expectancy [49]. Our care model reduced unnecessary medical services and saved medical resources. Polypharmacy and the high frequency of outpatient clinic visits remain serious problems that consume medical resources in our society. A change in health care policy and intervention can improve polypharmacy problems, as evidenced by Japan’s experience and in other studies [50, 51].

This retrospective study has some limitations. There may be patient selection bias in that we did not stratify the patients according to the number of comorbidities. The variation in different comorbidities could affect the amount of medical utilization. The included patients came from only one hospital. However, we selected patients from other internal medicine departments who were similar in age and comorbidities to be the control group and were matched by age and sex. This statistical method used in this study reduced the selection bias. Future studies of the geriatric integrated outpatient clinic approach may focus on its role in caring for patients with multiple comorbidities and for the oldest people and in providing late life care.

## Conclusion

The geriatric integrated outpatient clinic reduced medical costs, the number of drugs prescribed, and the frequency of outpatient clinic visits, emergency department visits and hospitalizations in a two-year follow-up of older patients with complicated conditions. The effect was much better in the second year. This model can provide a possible solution for older people with high medical utilization. Geriatric integrated outpatient care provides a comprehensive treatment plan and connections to social resources for older people, thereby reducing the utilization of medical resources. Geriatric health policy may encourage older people with high medical utilization to seek geriatric integrated outpatient care rather than visiting multiple specialists.

## Supplementary information

**Additional file 1: Table S1.** Demographics of patients aged **≥**80 years. **Table S2.** Demographics of the patients with a Charlson comorbidity index ≧ 2. **Table S3.** Health care utilization by patients aged ≧80 years. **Table S4.** Health care utilization by patients with a Charlson comorbidity index≧2. **Table S5.** Change in health care utilization by patients aged ≧80 years. **Table S6.** Change in health care utilization by patients with a Charlson comorbidity index ≧2

## Data Availability

All data generated or analysed during this study are included in this article and its supplementary information files.

## Abbreviations

Geri-OPD: Geriatric outpatient clinic
non-Geri-OPD: Non-geriatric outpatient clinics
CCI: Charlson comorbidity index
CGA: Comprehensive geriatric assessment
IQR: Interquartile range
