# Supplementary material for: The influence of integrated geriatric outpatient clinics on the health care utilization of older people
Source: BMC Geriatr. 2020 Oct 2;20:379. doi: 10.1186/s12877-020-01782-7 (PMC7531091; doi:10.1186/s12877-020-01782-7)
Supplement: Supplementary file 1 — Additional file 1: Table S1. Demographics of patients aged ≥80 years. Table S2. Demographics of the patients with a Charlson comorbidity index ≧ 2. Table S3. Health care utilization by patients aged ≧80 years. Table S4. Health care utilization by patients with a Charlson comorbidity index≧2. Table S5. Change in health care utilization by patients aged ≧80 years. Table S6. Change in health care utilization by patients with a Charlson comorbidity index ≧2 [file 12877_2020_1782_MOESM1_ESM.docx]

**Table S1.** Demographics of patients aged ≥80 years

|  | Total | Geri-OPD | Non-Geri-OPD | P |
| --- | --- | --- | --- | --- |
| Number, n (%) | 3628 (100) | 712 (19.6) | 2916 (80.4) |  |
| Sex |  |  |  | 0.99 |
| Female, n (%) | 2039 (56.2) | 400 (56.2) | 1639 (56.2) |  |
| Male, n (%) | 1589 (43.8) | 312 (43.8) | 1277 (43.8) |  |
| Age, mean ± standard deviation (SD) |  | 85.67 ± 4.17 | 85.60 ± 4.17 | - |
| Charlson comorbidity index (CCI) |  |  |  | <0.01 |
| CCI = 0, n (%) | 1467 (40.4) | 255 (35.8) | 1212 (41.6) |  |
| CCI = 1, n (%) | 748 (20.6) | 141 (19.8) | 607 (20.8) |  |
| CCI ≥ 2, n (%) | 1413 (38.9) | 316 (44.4) | 1097(37.6) |  |
| Medical problems, n (%) |  |  |  |  |
| Acute myocardial infarction | 132(3.6) | 24 (3.4) | 108 (3.7) | 0.67 |
| Congestive heart failure | 293 (8.1) | 72 (10.1) | 221 (7.6) | 0.03 |
| Peripheral vascular disease | 72 (2.0) | 19 (2.7) | 53 (1.8) | 0.14 |
| Cerebrovascular accident | 849 (23.4) | 159 (22.3) | 690 (23.7) | 0.45 |
| Dementia | 554 (15.3) | 107(15) | 447 (15.3) | 0.84 |
| Pulmonary disease | 434 (12.0) | 140 (19.7) | 294 (10.1) | <0.01 |
| Peptic ulcer | 469 (13.0) | 104 (14.6) | 365 (12.5) | 0.14 |
| Liver disease | 77 (2.1) | 16 (2.2) | 61 (2.1) | 0.80 |
| Diabetes mellitus | 674 (18.6) | 159 (22.3) | 515 (17.7) | <0.01 |
| Diabetes mellitus complications | 308 (8.5) | 67 (9.4) | 241 (8.3) | 0.33 |
| Renal disease | 453 (12.5) | 103 (14.5) | 350 (12) | 0.07 |
| Annual outpatient department visits (mean ± SD) |  | 68.21 ± 51.60 | 36.36 ± 47.75 | <0.01 |
| Annual emergency room visits (mean ± SD) |  | 2.44 ± 7.93 | 1.07 ± 3.20 | <0.01 |
| Number of drugs (mean ± SD) |  | 23.32 ± 5.86 | 16.21 ± 12.38 | <0.01 |
| Annual hospitalizations (mean ± SD) |  | 0.60 ± 1.08 | 0.25 ±0.67 | <0.01 |
| Length of hospital stay (days/year) |  | 7.96 ± 19.11 | 2.76 ± 8.91 | <0.01 |
| Cost of each clinic visit (log NTD, mean ± SD) |  | 7.41 ± 1.09 | 5.02 ± 3.47 | <0.01 |
| Cost of each hospitalization (log NTD, mean ± SD) |  | 3.86 ± 5.21 | 1.83 ± 4.08 | <0.01 |
| Cost of annual outpatient care (log NTD, mean ± SD) |  | 520.9 ± 407.4 | 272.4 ± 392.1 | <0.01 |
| Cost of annual hospitalizations (log NTD, mean ± SD) |  | 6.55 ± 1.66 | 2.72 ± 7.31 | <0.01 |

Abbreviation: NTD: New Taiwan dollars

**Table S2.** Demographics of the patients with a Charlson comorbidity index ≥2

|  | Total | Geri-OPD | Non-Geri-OPD | P |
| --- | --- | --- | --- | --- |
| Number, n (%) | 2326 (100) | 529 (22.7) | 1797 (77.3) |  |
| Sex |  | | | 0.63 |
| Female, n (%) | 1213 (52.15) | 271 (51.2) | 942 (52.4) |  |
| Male, n (%) | 1113 (47.85) | 258 (48.8) | 855 (47.6) |  |
| Age, mean ± standard deviation (SD) | 81.61 ± 6.28 | 81.44 ± 6.17 | 81.66 ± 6.32 | 0.48 |
| Medical problems, n (%) |  | | | |
| Acute myocardial infarction | 205 (8.8) | 40 (7.6) | 165 (9.2) | 0.25 |
| Congestive heart failure | 377 (16.2) | 98 (18.5) | 279 (15.5) | 0.10 |
| Peripheral vascular disease | 99 (4.3) | 27 (5.1) | 72 (4) | 0.27 |
| Cerebrovascular accident | 979 (42.1) | 218 (41.2) | 761 (42.3) | 0.64 |
| Dementia | 526 (22.6) | 123 (23.3) | 403 (22.4) | 0.69 |
| Pulmonary disease | 495 (21.3) | 167 (31.6) | 328 (18.3) | <0.01 |
| Peptic ulcer | 644 (27.7) | 160 (30.2) | 484 (26.9) | 0.13 |
| Liver disease | 139 (6.0) | 34 (6.4) | 105 (5.8) | 0.62 |
| Diabetes mellitus | 995 (42.8) | 249 (47.1) | 746 (41.5) | 0.02 |
| Diabetes mellitus complications | 595 (25.6) | 135 (25.5) | 460 (25.6) | 0.97 |
| Renal disease | 741 (31.9) | 157 (29.7) | 584 (32.5) | 0.22 |
| Depression | 139 (6.0) | 41 (7.8) | 98 (5.5) | 0.05 |
| Annual outpatient department visits (mean ± SD) |  | 84.91 ± 55.38 | 62.93 ± 63.02 | <0.01 |
| Annual emergency room visits (mean ± SD) |  | 3.14 ± 9.29 | 1.45 ± 4.21 | <0.01 |
| Number of drugs (mean ± SD) |  | 24.05 ± 4.86 | 21.69 ± 10.67 | <0.01 |
| Annual hospitalizations (mean ± SD) |  | 0.75 ± 1.19 | 0.33 ± 0.76 | <0.01 |
| Length of hospital stay (days/year) |  | 10.06 ± 21.29 | 3.85 ± 11.63 | <0.01 |
| Cost of each clinic visit (log NTD, mean ± SD) |  | 7.61 ± 0.66 | 6.45 ± 2.62 | <0.01 |
| Cost of each hospitalization (log NTD, mean ± SD) |  | 4.57 ± 5.38 | 2.36 ± 4.50 | <0.01 |
| Cost of annual outpatient care (log NTD, mean ± SD) |  | 652.5 ± 437.9 | 480.2 ± 557.3 | <0.01 |
| Cost of annual hospitalizations (log NTD, mean ± SD) |  | 8.18 ± 12.94 | 3.63 ± 8.41 | <0.01 |

Abbreviation: NTD: New Taiwan dollars

**Table S3.** Health care utilization by patients aged ≥80 years

|  | First year | | | Second year | | |
| --- | --- | --- | --- | --- | --- | --- |
|  | Geri-OPD  (N = 712) | Non-Geri-OPD  (N = 2916) | P | Geri-OPD  (N = 694) | Non-Geri-OPD  (N = 2827) | P |
| Annual outpatient department visits (mean ± SD) | 50.46 ± 57.22 | 40.69 ± 49.90 | <0.01 | 37.78 ± 53.02 | 35.27 ± 45.39 | 0.25 |
| Annual emergency room visits (mean ± SD) | 2.84 ± 7.25 | 1.17 ± 3.60 | <0.01 | 1.26 ± 3.55 | 1.07 ± 4.49 | 0.25 |
| Number of drugs (mean ± SD) | 17.52 ± 10.98 | 17.24 ± 12.23 | 0.54 | 15.78 ± 12.46 | 17.07 ± 12.32 | 0.01 |
| Annual hospitalizations (mean ± SD) | 0.75 ± 1.03 | 0.27 ± 0.73 | <0.01 | 0.27 ± 0.61 | 0.26 ± 0.68 | 0.71 |
| Length of hospital stay (days/year) | 9.59 ± 17.47 | 3.15 ± 10.86 | <0.01 | 2.69 ± 7.78 | 2.77 ± 9.75 | 0.80 |
| Cost of each clinic visit (log NTD, mean ± SD) | 6.14 ± 2.83 | 5.26 ± 3.35 | <0.01 | 4.85 ± 3.47 | 5.14 ± 3.40 | 0.04 |
| Cost of each hospitalization (log NTD, mean ± SD) | 5.20 ± 5.40 | 1.87 ± 4.10 | <0.01 | 2.14 ± 4.30 | 1.81 ± 4.03 | 0.07 |
| Cost of annual outpatient care (log NTD, mean ± SD) | 377.1 ± 465.3 | 304.0 ± 402.7 | 0.01 | 280.7 ± 436.5 | 262.4 ± 358.4 | 0.30 |
| Cost of annual hospitalizations (log NTD, mean ± SD) | 8.05 ± 11.15 | 2.96 ± 7.97 | <0.01 | 2.83 ± 6.50 | 2.76 ± 7.40 | 0.80 |

Abbreviation: NTD: New Taiwan dollars

**Table S4.** Health care utilization by patients with a Charlson comorbidity index ≥2

|  | First year | | | Second year | | |
| --- | --- | --- | --- | --- | --- | --- |
|  | Geri-OPD  (N = 529) | Non-Geri-OPD  (N = 1797) | P | Geri-OPD  (N = 512) | Non-Geri-OPD  (N = 1745) | P |
| Annual outpatient department visits (mean ± SD) | 77.36 ± 62.35 | 72.96 ± 59.79 | 0.14 | 56.56 ± 61.62 | 57.36 ± 56.15 | 0.79 |
| Annual emergency room visits (mean ± SD) | 3.77 ± 8.31 | 1.97 ± 5.30 | <0.01 | 2.04 ± 4.79 | 1.31 ± 5.10 | 0.01 |
| Number of drugs (mean ± SD) | 22.56 ± 6.95 | 24.27 ± 6.40 | <0.01 | 19.94 ± 10.62 | 22.37 ± 9.46 | <0.01 |
| Annual hospitalizations (mean ± SD) | 1.00 ± 1.12 | 0.48 ± 0.97 | <0.01 | 0.37 ± 0.74 | 0.31 ± 0.73 | 0.07 |
| Length of hospital stay (days/year) | 12.59 ± 19.23 | 5.63 ± 15.02 | <0.01 | 3.94 ± 9.69 | 3.19 ± 10.56 | 0.13 |
| Cost of each clinic visit (log NTD, mean ± SD) | 7.26 ± 1.24 | 7.32 ± 1.11 | 0.27 | 6.08 ± 2.87 | 6.65 ± 2.46 | 0.01 |
| Cost of each hospitalization (log NTD, mean ± SD) | 6.56 ± 5.29 | 3.16 ± 4.93 | <0.01 | 2.80 ± 4.73 | 2.20 ± 4.32 | 0.01 |
| Cost of annual outpatient care (log NTD, mean ± SD) | 581.0 ± 513.0 | 553.0 ± 521.1 | 0.28 | 425.5 ± 511.3 | 431.1 ± 470.5 | 0.82 |
| Cost of annual hospitalizations (log NTD, mean ± SD) | 10.75 ± 12.13 | 5.24 ± 10.63 | <0.01 | 3.97 ± 7.81 | 3.26 ± 7.81 | 0.06 |

Abbreviation: NTD: New Taiwan dollars

**Table S5.** Change in health care utilization by patients aged ≥80 years

|  | First year | | | Second year | | |
| --- | --- | --- | --- | --- | --- | --- |
|  | Geri-OPD  (N = 712) | Non-Geri-OPD  (N = 2916) | P | Geri-OPD  (N = 694) | Non-Geri-OPD  (N = 2827) | P |
| Annual outpatient department visits (mean ± SD) | -17.75 ± 51.94 | 4.33 ± 30.66 | <0.01 | -12.69 ± 32.76 | -5.42 ± 36.51 | <0.01 |
| Annual emergency room visits (mean ± SD) | 0.39 ± 6.93 | 0.10 ± 4.02 | 0.28 | -1.58 ± 6.74 | -0.09 ± 5.14 | <0.01 |
| Number of drugs (mean ± SD) | -5.79 ± 10.87 | 1.02 ± 10.44 | <0.01 | -1.74 ± 8.91 | -0.17 ± 10.40 | <0.01 |
| Annual hospitalizations (mean ± SD) | 0.14 ± 1.26 | 0.02 ± 0.90 | 0.02 | -0.48 ± 1.09 | -0.02 ± 0.89 | <0.01 |
| Length of hospital stay (days/year) | 1.63 ± 21.25 | 0.39 ± 12.91 | 0.14 | -6.90 ± 17.89 | -0.38 ± 12.78 | <0.01 |
| Cost of each clinic visit (log NTD, mean ± SD) | -1.27 ± 2.85 | 0.24 ± 3.36 | <0.01 | -1.30 ± 3.36 | -0.11 ± 3.41 | <0.01 |
| Cost of each hospitalization (log NTD, mean ± SD) | 1.34 ± 6.69 | 0.04 ± 5.30 | <0.01 | -3.06 ± 6.37 | -0.06 ± 5.25 | <0.01 |
| Cost of annual outpatient care (log NTD, mean ± SD) | -143.9 ± 423.3 | 31.54 ± 252.2 | <0.01 | -96.37 ± 262.2 | -41.53 ± 293.2 | <0.01 |
| Cost of annual hospitalizations (log NTD, mean ± SD) | 1.49 ± 13.54 | 0.24 ± 9.80 | 0.02 | -5.22 ± 11.71 | -0.20 ± 9.65 | <0.01 |

Abbreviation: NTD: New Taiwan dollar**s**

**Table S6.** Change in health care utilization by patients with a Charlson comorbidity index ≥2

|  | First year | | | Second year | | |
| --- | --- | --- | --- | --- | --- | --- |
|  | Geri-OPD  (N = 529) | Non-Geri-OPD  (N = 1797) | P | Geri-OPD  (N = 512) | Non-Geri-OPD  (N = 1745) | P |
| Annual outpatient department visits (mean ± SD) | -7.56 ± 55.95 | 10.04 ± 39.85 | <0.01 | -20.80 ± 43.92 | -15.61 ± 44.58 | 0.01 |
| Annual emergency room visits (mean ± SD) | 0.63 ± 8.17 | 0.52 ± 5.24 | 0.77 | -1.73 ± 8.15 | -0.67 ± 6.40 | 0.01 |
| Number of drugs (mean ± SD) | -1.49 ± 7.34 | 2.58 ± 8.96 | <0.01 | -2.62 ± 8.72 | -1.89 ± 7.88 | 0.08 |
| Annual hospitalizations (mean ± SD) | 0.24 ± 1.33 | 0.15 ± 1.09 | 0.13 | -0.62 ± 1.19 | -0.18 ± 1.07 | <0.01 |
| Length of hospital stay (days/year) | 2.53 ± 23.56 | 1.78 ± 17.28 | 0.50 | -8.65 ± 19.95 | -2.45 ± 15.91 | <0.01 |
| Cost of each clinic visit (log NTD, mean ± SD) | -0.35 ± 1.31 | 0.87 ± 2.57 | <0.01 | -1.18 ± 2.86 | -0.78 ± 2.48 | 0.01 |
| Cost of each hospitalization (log NTD, mean ± SD) | 1.99 ± 6.59 | 0.80 ± 6.04 | 0.01 | -3.76 ± 6.38 | -0.96 ± 5.96 | <0.01 |
| Cost of annual outpatient care (log NTD, mean ± SD) | -71.44 ± 460.10 | 72.82 ± 343.00 | <0.01 | -155.6 ± 346.4 | -121.9 ± 363.8 | 0.06 |
| Cost of annual hospitalizations (log NTD, mean ± SD) | 2.58 ± 14.43 | 1.60 ± 11.98 | 0.16 | -6.78 ± 12.87 | -1.98 ± 11.54 | <0.01 |

Abbreviation: NTD: New Taiwan dollars
